# The Clinical and Steroid-Free Remission of Fecal Microbiota Transplantation to Patients with Ulcerative Colitis: A Meta-Analysis

**DOI:** 10.1155/2019/1287493

**Published:** 2019-04-15

**Authors:** Wai Ching Lam, Chen Zhao, Wen Juan Ma, Liang Yao

**Affiliations:** ^1^School of Chinese Medicine Hong Kong Baptist University Hong Kong, China; ^2^Evidence-based Medicine Center, School of Basic Medical Science, Lanzhou University, China

## Abstract

**Background and Purpose:**

Since the first case of fecal microbiota transplantation for the treatment of ulcerative colitis was described in the year 1989, there have been an increment of case reports, case series, cohort studies, and randomized controlled trials (RCTs). In this study, we were going to investigate general clinical remission, clinical response, and steroid-free remission of fecal microbiota transplantation.

**Methods:**

We searched Ovid Medline, Ovid EMBASE, and Cochrane Library, focusing prospective studies including randomized controlled trials and cohort studies. The outcomes were clinical remission, clinical response, steroid-free remission, and serious adverse events. We used RevMan 5.3 software for meta-analyses.

**Key Results:**

A total of 4 RCTs and 2 cohort studies (340 cases from 5 countries) were included. We found that FMT might be more effective than placebo on clinical remission (OR, 3.85 [2.21, 6.7]; *P* < 0.001; *I*^2^ = 0%) and clinical response (OR, 2.75 [1.33, 5.67]; *P* = 0.006; *I*^2^ = 49%), but no statistical difference on steroid-free remission (OR, 2.08 [0.41, 10.5]; *P* = 0.37; *I*^2^ = 69%) and serious adverse events (OR, 2.0 [0.17, 22.97]; *P* = 0.44; *I*^2^ = 0%).

**Conclusions and Inferences:**

Fecal microbiota transplantations were associated with significant clinical remission and response in ulcerative colitis patients while there was no significant difference found between FMT and placebo in steroid-free remission. Moreover, a common consensus on the route, volume, timing, preferred donor characteristics, and frequency of fecal administration is necessary to achieve remission.

## 1. Introduction

As a major subtype of inflammatory bowel disease (IBD), ulcerative colitis (UC) is a chronic, relapsing, and remittable immunologically mediated disease affecting the colonic mucosa. With the increasing incidence rate around the world [1], UC is still without a medical cure and commonly requires a lifetime of care and medication. Being a disease with unknown etiology affects, an estimated 1.5 million Americans, 2.2 million people in Europe, and several hundred thousands more worldwide [2, 3], UC increases the burden of therapy, hospitalizations, surgery, health-related quality of life, economic productivity, and social functioning [4].

We aim to induce and maintain remission and reduce the risk of complications. Conventional treatments, including aminosalicylate, corticosteroid, thiopurine, and immunosuppressant, for UC are based on the severity of disease and patient preference [5–7]. However, these treatments often have limitations with the severity of UC and come with serious side effects [8]. In recent years, research on new interventions for microflora has been promoted due to a report that intestinal microbiota plays a key role in UC [9, 10].

Fecal microbiota transplantation (FMT) is the transfer of stool from a healthy donor into the colon of a patient in order to change the microbial communities. Since multiple studies have demonstrated differences in the composition of the gut microbiota between patients with UC and healthy individuals, plus in recent decades, genome-wide associated studies and other genetic analyses showed that intestinal microbiota plays roles in aberrant immune response in IBD [10–13]. As a result, FMT is hypothesized as a potential novel treatment for patients with UC [14].

After the first case of fecal microbiota transplantation for the treatment of ulcerative colitis was described in the year 1989 [15], there has been an increment of case reports, case series, cohort studies, and randomized controlled trials (RCTs). Currently, as a recommended therapy for moderate to severe UC [8, 16], steroid-dependent ulcerative colitis became a new issue with various side effects on the patients [17]. Although most of steroid-dependent UC generally is a serious condition, with risks of long-term steroid therapy including osteoporosis, aseptic joint necrosis, metabolic changes, and psychological disturbances, steroids are treated effective in inducing remission but not acceptable to maintain remission [8]. Therefore, there is a necessity to focus on the value of steroid-free FMT, to figure out the independent treatment effects of FMT as a potential solution for UC. Furthermore, clinically, steroid-free FMT is especially important for the management of patients with steroid-refractory UC [18].

As a result, in order to investigate both general and steroid-free FMT treatment effects, we decided to carry out this systematic review with meta-analysis by evidence from controlled studies.

## 2. Methods

### 2.1. Searching Strategy

This systematic review with meta-analyses was conducted in line with the recommendations from the Preferred Reporting Items for Systematic Reviews and Meta-Analysis (PRISMA) statement [19]. A searching strategy developed according to key terms was referred to Table 1. One reviewer (WCL) searched Ovid Medline, Ovid EMBASE, and Cochrane Library from inception to the 30th of November 2018. There was no restriction on language or publication status.

### 2.2. Inclusion Criteria

The following inclusion criteria were applied: (1) prospective study including RCTs, and cohort studies, (2) patients who are diagnosed with UC; (3) patients who received any FMT type or combination therapies compared with placebo.

### 2.3. Data Extraction

**Figure 1:**
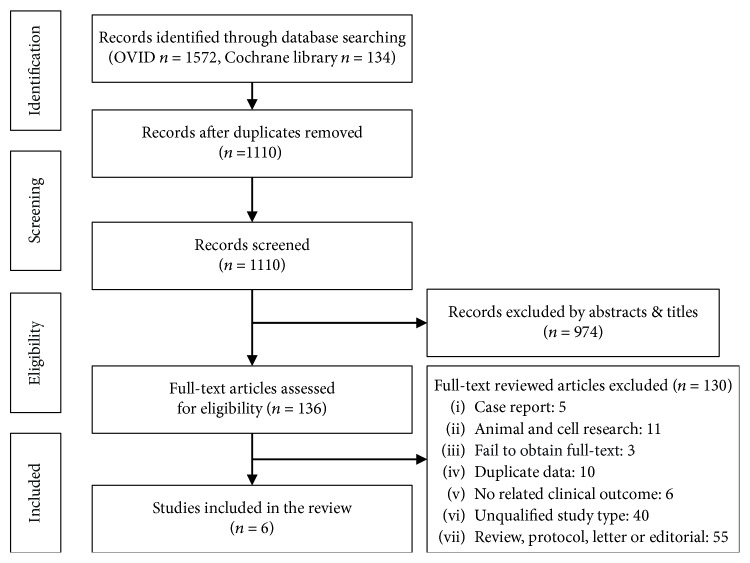
Workflow.

EndNote X8 software was used to manage the literatures searched from the database for omitting duplicates (Figure 1). Two authors (CZ and WJM) independently identified abstracts, titles, and full texts of articles for eligibility. Disputes were resolved by discussion with another author (WCL).

Two review authors (CZ and WJM) independently extract data from the included studies. The following information will be extracted using an Excel 2017 software data form: general information (title, authors, country of study, funding, year of publication, and registry number (if any)); details of study (aim, design type, and inclusion and exclusion criteria); study population (age, sex, sample size, number for analysis, and severity of UC); FMT characteristics (type of FMT, dose, pretreatment, and combination therapies); outcome (primary and secondary outcomes, time points, response rate, and method of response assessment).

### 2.4. Assessing Methodological Quality

Two independent authors (WJM and LY) assessed article quality according to the Cochrane risk of bias tool for RCTs and the Newcastle-Ottawa Scale (NOS) for cohort studies [20, 21].

### 2.5. Statistical Analysis

RevMan 5.3 software was used for statistical analyses. Meta-analysis was conducted in subgroups about clinical remission, clinical response, steroid-free remission, and serious adverse events (SAE). Dichotomous outcomes used a pooled odds ratio (OR) with 95% confidence interval (CI) to estimate the report effect. Heterogeneity was assessed using the *I*-squared statistic, and the *I*-squared value > 50% was considered to be indicative of substantial heterogeneity. The fixed effects model was used to combine dichotomous data if the *I*-squared value < 50%. The random effects model was used if the *I*-squared value > 50%.

### 2.6. Publication Bias

Egger's test would be performed to explore publication bias when applicable (the number of included studies no less than 10).

## 3. Results

### 3.1. Characteristics of Included Studies

Four RCTs [22–25] with 277 participants and two cohort [26, 27] studies with 63 participants were included for analysis. Two of the trials came from Australia, and the rest two were from Canada and Netherlands, respectively. The two cohorts were from Japan and Australia. The route of giving FMT varied among studies, including transplantation, retention enema, nasoduodenal tube, infusion, colonoscopy, and endoscopy. Participants received frozen FMT in three studies [22, 25, 27], fresh FMT in two studies [24, 26], and both frozen and fresh FMT in one trial [23]. More characteristics of included studies are summarized in Table 2.

### 3.2. Risk of Bias Assessment

Risk assessment of included random controlled trials is shown in Table 3. The 4 included RCTs got high quality in random sequence generation and selection reporting. Two RCTs were at unclear quality in allocation concealment, and one was at low quality at incomplete outcome data. One trial got unclear quality in blinding of participants, and 3 trials got unclear quality in blinding of outcome assessment. Risk bias assessment of cohort studies can be found in Table 4; in general, the quality of the 2 cohort studies is low.

### 3.3. Clinical Remission

**Figure 2:**
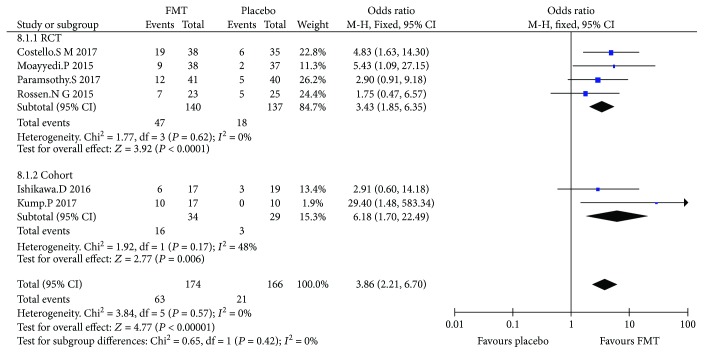
Meta-analysis of clinical remission in patients undergoing FMT versus placebo; *P* < 0.05.

Four RCTs and 2 cohort studies (340 cases in total) reported the clinical remission compared to FMT with placebo. The average clinical remission of FMT was 33.6% (23.7-50%) in the RCT group and 47% (35.3-58.8%) in the cohort. Meta-analysis results showed that there was a significant difference between FMT and placebo (Figure 2), the overall OR (95% CI) of RCTs was 3.43 (1.85, 6.35), and that of the cohort study was 6.18 (1.7, 22.49); the results also showed that there was no or low heterogeneity among RCTs (*I*^2^ = 0%, *P* = 0.62) and low heterogeneity among cohort studies (*I*^2^ = 48%, *P* = 0.17).

The total overall OR of RCTs and cohort studies was 3.85 (2.21, 6.7); there was a statistical difference between FMT and placebo and no or low heterogeneity among all studies (*I*^2^ = 0%, *P* = 0.57).

### 3.4. Clinical Response

**Figure 3:**
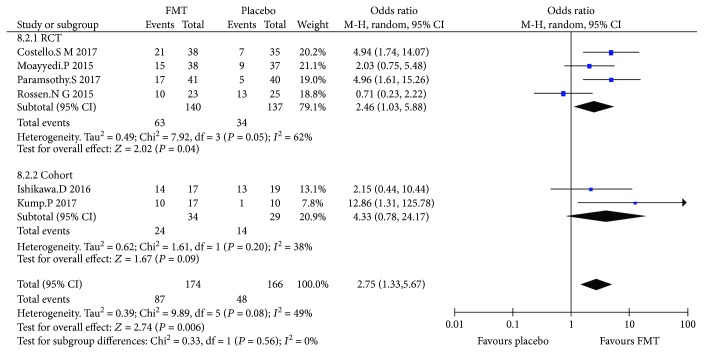
Meta-analysis of clinical response in patients undergoing FMT versus placebo; *P* < 0.05.

Four RCTs and two cohort studies (340 cases in total) reported the clinical response compared to FMT with placebo. The average clinical response of FMT was 45% (39.5-55.3%) in RCT and 70.6% (58.8-82.4%) in cohort. The pooled results showed that there was a significant difference between FMT and placebo of RCTs, but no significant difference in cohort studies (Figure 3), the overall OR (95% CI) of RCTs was 2.46 (1.03, 5.88), and that of cohort studies was 4.33 (0.78, 24.17); the results also showed that there was high heterogeneity among RCTs (*I*^2^ = 62%, *P* = 0.05) and low heterogeneity among cohort studies (*I*^2^ = 38%, *P* = 0.20).

The total overall OR of RCTs and cohort studies was 2.75 (1.33, 5.67), there was a statistical difference between FMT and placebo, and there was low heterogeneity among all studies (*I*^2^ = 49%, *P* = 0.08).

### 3.5. Steroid-Free Remission

**Figure 4:**
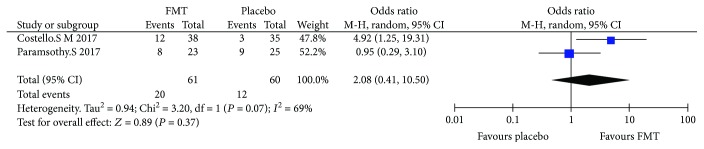
Meta-analysis of steroid-free remission in patients undergoing FMT versus placebo; *P* = 0.37.

Two RCTs (121 cases in total) reported the steroid-free remission. The average steroid-free remission of FMT was 32.7% (31.6-34.8%) in RCT. The pooled results showed that there was no significant difference between FMT and placebo in steroid-free remission (Figure 4), the OR was 2.08 (0.41, 10.5), and the results also showed that there was high heterogeneity among RCTs (*I*^2^ = 69%, *P* = 0.07); then, we used a random effects model.

### 3.6. Serious Adverse Events

**Figure 5:**
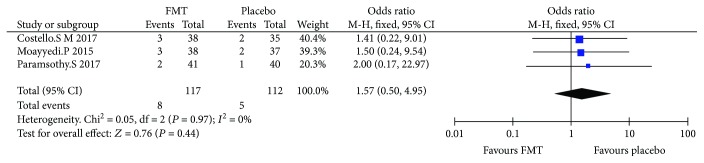
Meta-analysis of patients with serious adverse events undergoing FMT versus placebo; *P* = 0.44.

Three RCTs (229 cases in total) reported the patients with SAE. The average rate of serious adverse events was 6.8% (4.9-7.9%) in RCT. Meta-analysis showed that there was no significant difference between FMT and placebo (Figure 5), the OR was 2.0 (0.17, 22.97), and the results also showed that there was low or no heterogeneity among RCTs (*I*^2^ = 0%, *P* = 0.97).

### 3.7. Publication Bias

Since the number of studies included was less than 10, no Egger test was performed.

## 4. Discussion

This systematic review and meta-analysis showed that in FMT studies, there are differences in the significance between clinical remission and clinical response on the patients receiving FMT. The pooled analysis demonstrated, especially in RCTs, that FMT were significantly associated with improved clinical remission and clinical response compared to placebo. These results are consistent with those of a recent systematic review and meta-analysis of RCTs by Costello et al. and Narula et al. [28, 29], who found significant association between FMT and remission for active UC. However, they did not include steroid-free remission which can be viewed as an alternative choice in terms of clinical decisions. In our study, for the pooled analysis of steroid-free remission from 2 RCTs [22, 25], the results showed that there was no significant difference between FMT and placebo in steroid-free remission. However, the result might need to be confirmed by more studies, as significant heterogeneity among studies, bias for selecting original steroid-dependent patients, and the mandatory steroid wean was demanding resulting in several withdrawing from the studies.

Furthermore, unlike the pervious systematic reviews mentioned [28, 29], we included prospective cohort studies with parallel control groups into pooled analyses, to compare with and investigate the difference of results from RCTs for clinical remission and response. For clinical remission, we found that the pooled results from RCTs and cohort studies pointed to the same orientation. However, as there was different situation for clinical response, we tended to support pooled result from RCTs due to the strength of evidence based on the study design and nature of the research question.

Although the insignificant serious adverse effects reported in patients with UC supported its safety for application, the elephant in the room is not only for our included studies, but there is no common consensus on the route, volume, timing, preferred donor characteristics, and frequency of fecal administration necessary to achieve remission [30]. As a substantial proportion of UC patients are associated with ongoing widespread infection, mental health or behavioral problems, certain underlying physical conditions, or taking another medication that may interact with steroids, studies in the efficacy and safety of steroid-free FMT will become clinically helpful. Further, more research is required to explore standard methods and treatment protocols of FMT for different subtypes of UC patients.

For the route of FMT, in our included studies, all the transplantations are through fresh or frozen feces. In general, frozen feces have advantages over fresh in aspects of preparation, storage, monitoring, and delivering FMT at centers that do not have on-site laboratory facilities [31, 32]. Without direct evidence on UC, multistudies of Clostridium difficile infection patients demonstrated that compared to fresh FMT, frozen FMT had equal effects and risk of adverse events [32–36]. Moreover, based on identification of specific bacterial species by real-time quantitative PCR (qPCR), frozen and lyophilized FMT products were stored up to 7 months without losing microbiota composition [37].

It is important to mention that all studies included have undergone pretreatment with antibiotics. As highlighted by other reviewers [38, 39], one of the major factors that may optimize the FMT treatment effect is to clean up original microflora in bowel prior to FMT as to provide rooms for new microbiota to function. In a FMT clinical trial with arm of antibiotics alone [26], plus a study carried out for further microbial analyses using a higher-resolution method to identify the colonization of key bacterial species [40], these findings preliminarily corroborated the hypothesis and serve as a basis for further investigations into the mechanisms of FMT and antibiotics.

Clinically, UC is a chronic disease without a pharmacological cure that usually requires regular, indefinite therapy to maintain remission [41]. FMT offers an option available to clinicians aiming to alter the intestinal immune system through restoring gut microbial diversity in recipients toward that of a healthy person as IBD is typically characterized by reduced microbial diversity [42–45]. Considering that most of steroid dependent UC generally are serious condition which might be not suitable for RCT, we also summarized results from large case series (N ≥ 10) to demonstrate a more complete picture. We found five large case series [17, 46–49] from databases and showed the characteristics and results in Table 5. We found the average clinical response and clinical remission was 50.4 (26.8-80%) and 15.2% (0 to 35%), respectively, no serious adverse event was reported, and none of these studies reported steroid-free remission outcome. The results from large-case series were consistent with the results from RCTs in clinical response but with lower clinical remission. However, the results in case series should be interpreted cautiously in clinical practice as potential selection bias might be existed.

Several limitations are encountered during this study. First, the number of clinical trials and cohort studies is limited. There is lack of large amount of patients with the same subtype and stage of UC; thus, the generalizability is limited. Second, the FMT and placebo in studies are prepared differently and with various dosages. Therefore, we could not effectively examine the dosage effect across the outcomes. Third, although the AE/SAE profile is an important factor for choosing treatment options, it was not possible to perform an analysis to deal with such a concern because AE/SAE are not fully reported in all included trials. Fourth, in the included trials, FMT can be independently applied or combined with other drugs as interventions; therefore, some of the therapeutic effects can be due to the interacted result between FMT and other components.

## 5. Conclusion

Fecal microbiota transplantations were associated with significant clinical remission and response in ulcerative colitis patients while there was no significant difference found between FMT and placebo in steroid-free remission. Moreover, a common consensus on the route, volume, timing, preferred donor characteristics, and frequency of fecal administration is necessary to achieve remission.

## Figures and Tables

**Table:** (a) Ovid Medline and Ovid EMBASE search strategy

1	Colitis.mp.
2	Exp colitis/
3	Proctosigmoiditis.mp.
4	Rectocolitis.mp.
5	Rectosigmoiditis.mp.
6	Haemorrhagic proctocolitis.mp.
7	Proctitis.mp.
8	Inflammatory bowel disease.mp.
9	Exp inflammatory bowel disease/
10	IBD.mp.
11	1 or 2 or 3 or 4 or 5 or 6 or 7 or 8 or 9 or 10
12	Fecal microbiota transplant^∗^.mp.
13	Faecal microbiota transplant^∗^.mp.
14	Fecal microbiome transplant^∗^.mp.
15	Fecal microflora transplant^∗^.mp.
16	Stool transplant^∗^.mp.
17	FMT.mp.
18	Fecal transfusion^∗^.mp.
19	Fecal bacteriotherap^∗^.mp.
20	12 or 13 or 14 or 15 or 16 or 17 or 18 or 19
23	11 and 20

**Table:** (b) Cochrane Library search strategy

#1	Colitis
#2	MeSH: [Colitis] explode all trees
#3	Proctosigmoiditis
#4	Rectocolitis
#5	Rectosigmoiditis
#6	Haemorrhagic proctocolitis
#7	Proctitis
#8	Inflammatory bowel disease
#9	MeSH: [inflammatory bowel disease] explode all trees
#10	IBD
#11	#1 or #2 or #3 or #4 or #5 or #6 or #7 or #8 or #9 or #10
#12	MeSH: [Fecal microbiota transplantation] explode all trees
#13	Fecal microbiota transplant^∗^
#14	Faecal microbiota transplant^∗^
#15	Fecal microbiome transplant^∗^
#16	Fecal microflora transplant^∗^
#17	Stool transplant^∗^
#18	FMT
#19	Fecal transfusion^∗^
#20	Fecal bacteriotherap^∗^
#21	#12 or #13 or #14 or #15 or #16 or #17 or #18 or #19 or #20
#22	#11 and #21

**Table 2:** Characteristics of the included studies.

Study	Type	Country	Sample	Severity of the disease	Route	Fresh/frozen	Placebo type
Costello et al. [22]	RCT	Australia	73	Mild-moderate active UC	Transplantation	Frozen	Autologous FMT
Moayyedi et al. [23]	RCT	Canada	75	Active UC	Retention enema	Fresh and frozen	Consisting of 50 mL water
Rossen et al. [24]	RCT	Netherlands	48	Mild to moderate active UC	Nasoduodenal tube	Fresh	Autologous fecal microbiota
Paramsothy et al. [25]	RCT	Australia	81	Active UC	Infusion	Frozen	Isotonic saline adding brown food colourant, odourant, and glycerol cryoprotectant
Ishikawa et al. [26]	COHORT	Japan	36	Mild-to-severe active UC	Colonoscopy	Fresh	AFM monotherapy
Kump et al. 2017 [27]	COHORT	Austria	27	Refractory ulcerative colitis, chronic active ulcerative colitis	Endoscopy	Frozen	Antibiotic treatment

Note: RCT: random controlled trials; COHORT: cohort studies; UC: ulcerative colitis; FMT: fecal microbiota transplantation; AFM: amoxicillin, fosfomycin, and metronidazole.

**Table 3:** Risk of bias assessment of included random controlled trials.

Study	Random sequence generation	Allocation concealment	Blinding of participants and personal	Blinding of outcome assessment	Incomplete outcome data	Selection reporting
Costello et al. [22]	Low risk	Unclear	Low risk	Unclear	Low risk	Low risk
Moayyedi et al. [23]	Low risk	Unclear	Unclear	Unclear	High risk	Low risk
Rossen et al. [24]	Low risk	Low risk	Low risk	High risk	Low risk	Low risk
Paramsothy et al. [25]	Low risk	Low risk	Low risk	Unclear	Low risk	Low risk

**Table 4:** Risk of bias assessment of included cohort studies.

Study	Item 1	Item 2	Item 3	Item 4	Item 5	Item 6	Item 7	Item 8
Kump et al. [27]	1	1	0	0	1	1	1	1
Ishikawa et al. [26]	1	1	0	0	1	1	1	1

Item 1: representativeness of the exposed cohort; Item 2: selection of the nonexposed cohort; Item 3: ascertainment of exposure; Item 4: demonstration that the outcome of interest was not present at the start of study; Item 5: comparability of cohorts on the basis of the design or analysis; Item 6: assessment of outcome; Item 7: was follow-up long enough for outcomes to occur; Item 8: adequacy of the follow-up of the cohort.

**Table 5:** Characteristics and results of large case series (*N* ≥ 10) of FMT in UC.

Study	Sample	Country	Age (year)	Severity	Route	Donor	Fresh/frozen	Clinical response	Clinical remission	Steroid-free remission	Serious adverse events	Follow-up (months)
Kunde et al. [46]	10	America	7-21	Mild-moderate	Colonoscopy	Family members or close friends	Fresh	6 (60%)	3 (30%)	NR	0	1
Cui et al. [17]	15	China	11-48	Moderate-severe	Gastroscope	Patients' healthy relatives or friends	Frozen	12 (80%)	4 (26.7%)	NR	0	4-72
Nishida et al. [47]	41	Japan	NR	Mild-moderate	Colonoscopy	Family members	Fresh	11 (26.8%)	0 (0)	NR	0	2
Wei et al. [48]	20	China	18-70	Mild-moderate	Colonoscopy	Healthy people	Fresh	13 (65%)	7 (35%)	NR	0	3
Zhang et al. [49]	19	China	19-60	Moderate-severe	Gastroscope	NR	Fresh	11 (57.9%)	2 (10.5%)	NR	0	≥3

NR: not reported.
